# An effective and considerable treatment of pilonidal sinus disease by laser ablation

**DOI:** 10.1007/s10103-023-03741-1

**Published:** 2023-03-01

**Authors:** Zhicheng Li, Lei Jin, Tianyun Gong, Kaijian Qin, Can Cui, Zhenyi Wang, Jiong Wu

**Affiliations:** Department of Coloproctology, Yueyang Hospital of Integrated Traditional Chinese and Western Medicine, Shanghai University of Traditional Chinese Medicine, Shanghai, 200437 China

**Keywords:** Sacrococcygeal pilonidal disease, Laser ablation, Surgical, Anorectal

## Abstract

The treatment of sacrococcygeal pilonidal disease (SPD) is still challenging. Although many non-surgical and surgical methods exist, no consensus has been reached on the best treatment. This study aimed to evaluate the efficacy of laser ablation using 1470‐nm radial diode laser fiber in treating SPD. We retrospectively studied the data of our 48 patients who operated on this technique between March 2019 and July 2022. All patients were treated with laser ablation using 1470‐nm radial diode laser fiber. The healing rate and recurrence rate, demographic and surgical data, postoperative pain, complications (wound infection, wound bleeding), the time of returning to regular work and life, and the time of wound healing were recorded. Postoperative pain was measured based on the visual analog scale (VAS) score. Postoperative follow-up was performed in the outpatient clinic every 1 week for 1 month. Among the 48 patients, 41 males and 7 females, with a mean age of 27.7 years (range 14–42), the healing rate was 100%, and the average healing time was 28.3 ± 5.5 days. Mean operative time was 15.5 ± 3.3 min. The recurrence rate was 2.1%. One patient relapsed 3 months after the operation. The patient underwent laser ablation again, and the sinus tract was closed. The median visual analog scale (VAS) score on the day of operation was 0(0,2). The median VAS score on the first, third, seventh, and fourteenth day after operation was 0(0,2), 0(0,1), 0(0,1), and 0(0,0), respectively. There was no wound infection or bleeding after the operation. The mean time to normal work/life was 7.1 ± 3.2 days. Almost all the patients felt very satisfied with the operation. Laser ablation using 1470‐nm radial diode laser fiber is effective in SPD treatment. It is associated with minor wounds and mild postoperative pain. It is a simple, safe, and minimally invasive technique and its clinical application for acute and chronic SPD in the absence of abscess is promising.

## Introduction

Sacrococcygeal pilonidal disease (SPD) refers to a subcutaneous infection that occurs in the gluteal sulcus of the sacrococcyx. Although the cause is not clear, it is believed to be acquired, and it is related to obesity, exuberant hair, and the deep cleft between the buttocks [1]. The incidence rate is highest among the Caucasian population; in Europe and the USA, its incidence is 26/100,000, most of the patients are aged between 15 and 30 years [2]. The incidence rate among males is 4 times higher than that among females [3]. The incidence rate in China is also increasing. The acute phase is characterized by sacral abscess, and during the chronic phase cyst formation or persistent sinus discharge may be observed. At present, no consensus exists regarding the best treatment choice, but conservative treatment can only control the symptoms, and most patients have to undergo operation [4]. Traditional surgery brings favorable success rates; it requires the removal of all diseased skin and subcutaneous tissue, including wide excision and healing with secondary intention or reconstructive (“flap”) techniques, which lead to a prolonged recovery period and a large wound [5]. In recent years, minimally invasive surgical methods for the treatment of SPD (such as endoscopic pilonidal sinus ablation or fibrin glue injection) have improved; wound injury and pain have been reduced, and the time for patients to return to normal life has been shortened [6–8].

Laser ablation using 1470‐nm radial diode laser fiber was first proposed by Wilhelm, which was first used to treat anal fistula with a cure rate of 82% [9]. The energy released by the annular laser at the end of the catheter makes the tissue in the fistula gasify and contract, closing the fistula. In 2016, Dessily et al. [10] used this technique for the first time in the treatment of pilonidal sinuses, with a success rate of 87.5% and a recurrence rate of only 2.9%, with mild postoperative pain and minor wounds. Subsequent studies have reported similar results [10–16]. Therefore, laser ablation closure may be the method of choice for the treatment of SPD.

To the best of our knowledge, we are the first institution to introduce Filac technology in China. On the basis of the certain efficacy of laser in the treatment of anal fistula, we tried to use laser to treat SPD in 2019. The wound healing rate was 100%, and the recurrence rate was only 6.7%. However, the number of patients included was relatively small, only 15 patients. Based on these encouraging data, this study further included patients who received SILAC® treatment in our hospital from March 2019 to July 2022 in order to evaluate the safety, effectiveness, and satisfactory results of the technique.

## Materials and methods

From March 2019 to July 2022, 48 consecutive patients with SPD were operated on by a surgeon with laser ablation. The operative technique and potential risks and complications of the procedure were explained to the patients. After a detailed explanation of the surgical techniques and potential risks, all patients signed an informed consent form before operation. All patients were examined by MRI or ultrasound before operation and diagnosed as SPD in order to exclude presacral tumors and other perianal diseases. Patients with SPD in the acute abscess stage were excluded from the present analysis.

### Technique

**Fig. 1:**
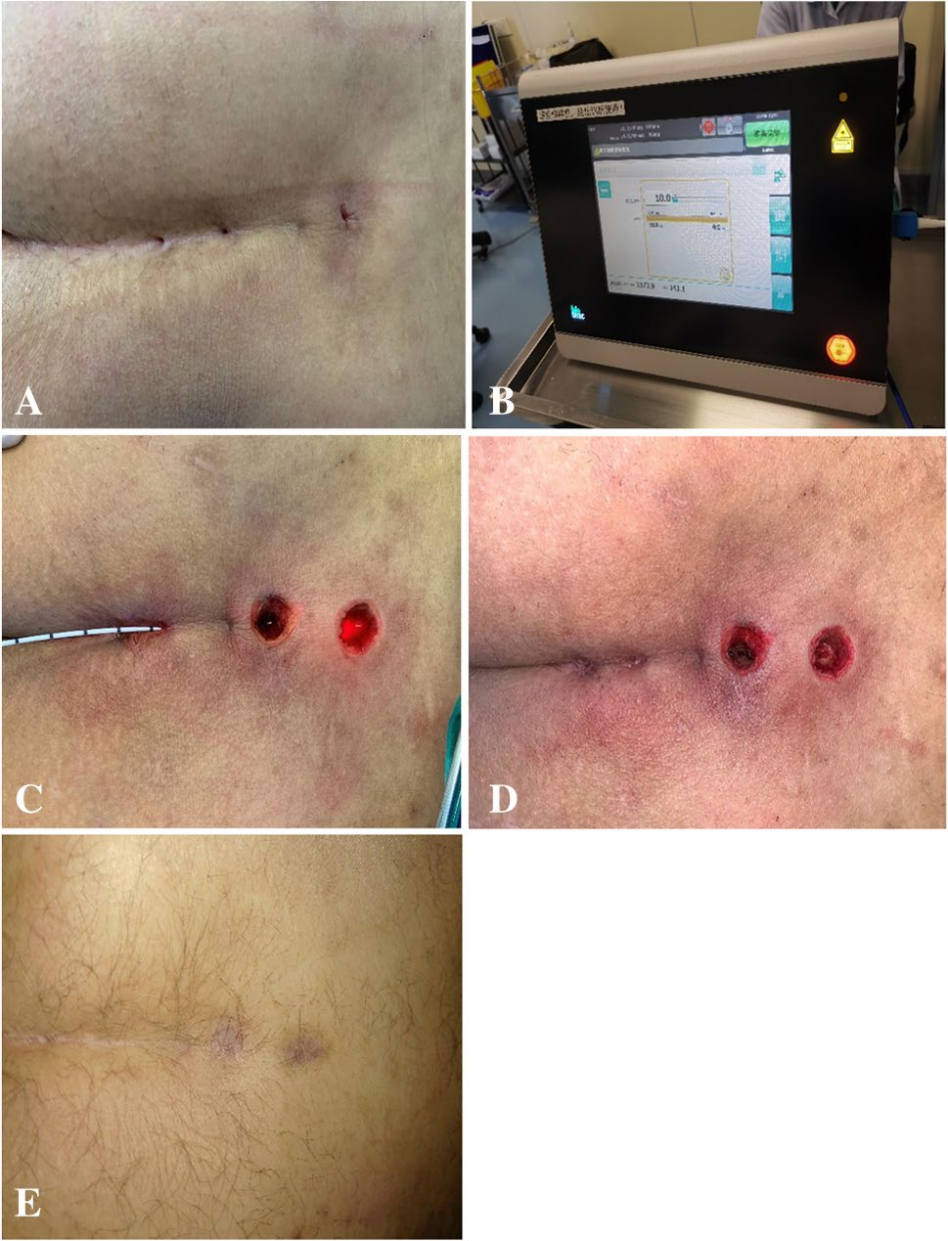
**A** The pits before the operation, **B** Biolitec device used for sinus laser treatment, **C** destruction of the tract with the laser, **D** after the operation, **E** successful healing at 1 year

All patients were treated with sacrocaudal skin preparation before operation. They were placed in the lateral recumbent position for easy anesthesia; strong tape is placed on the buttock to increase exposure. Propofol medium/long-chain fat emulsion was injected to induce and maintain intra-venous anesthesia (propofol was infused by target-controlled infusion, taking into account the age, height, and weight of the patient). Anesthesia was induced by 3 μg/mL and maintained by 2 μg/mL propofol. Then, a probe was used to evaluate the length and extent of the sinus from the pit (if there were multiple pits, the sinus tracts were evaluated separately). According to the size of the sinus infection area, a circular or oval incision was made to widen the existing sinus appropriately to ensure sufficient drainage. Mosquito clamps were used from the incision to clean the hair in the sinus tract. Then, the sinus tract was cleaned with a curette. The optical fiber (Leonardo Dual 45 Laser 980 nm/1470 nm, CeramOptec GmbH of Biolitec AG, Bonn, Germany, single radial laser fiber) was probed from the small concave side, under the guidance of the indicator light, along the sinus tract to the end. The laser power was 10 W and the wavelength was 1470 nm. The parcel was ablated between the sinus and pit by pulling the optical fiber at 1 mm/s. The situation before and after operation is shown in Fig. 1.

### Postoperative instructions

After hospitalization for several days, patients were instructed to clean the wound with iodophor every day, then press the tract, promote the discharge of secretions, and prevent premature closing of the skin. Topical use of antibiotic ointment. Instruct the patient to take painkillers if necessary. After discharge, the patient went to the outpatient clinic every week until the wound healed.

### Statistics

All statistical analyses were performed with the SPSS 25.0 statistical software. Values are presented as the mean ± standard deviation for data that were normally distributed or median and interquartile range for data that were not normally distributed for continuous variables and number (%) for categorical variables. The Kolmogorov–Smirnov test was used to inspect the normality and homogeneity of variance of all the data. For two-group comparison, *P* values were derived from the one-way Student *t*-test to determine differences between groups with normally distributed data and Mann–Whitney nonparametric test with other data. For all comparisons, *P* < 0.05 was considered statistically significant.

## Results

Baseline characteristics are presented in Table 1. A total of 41 males and 7 females were treated, with an average age of 27.7 years (range 14–42 years). All patients received regular telephone or outpatient follow-up. The median sinus length was 34 mm (interquartile range, 25.5–50.0 mm; range 15–100 mm). Thirteen patients had undergone incision and received drainage of sinus abscess, and 5 patients had undergone SPD-related surgery. The sinuses of 10 patients were in the acute stage in the absence of abscess (with empyema), and the others were in the chronic stage (no pus overflow and swelling). The median visual analog scale (VAS) score on the day of operation was 0(0,2). The median VAS score on the first, third, seventh, and fourteenth day after operation was 0(0,2), 0(0,1), 0(0,1), and 0(0,0), respectively. There was no wound infection or bleeding after operation. The average time of returning to normal work and life after operation was 7.1 ± 3.1 days. Almost all the patients felt very satisfied with the operation (Table 2). The wound healing rate was 100%, and the average wound healing time was 32.4 ± 5.4 days. The recurrence rate was 2.1%. There is no difference among diverse stage of SPD in healing and recurrence (Table 3). One patient, who was in the stage of inflammation before operation, relapsed 3 months postoperatively. In this patient, there was repeated local redness and swelling in the postoperative period and secretion overflow after ulceration. The patient underwent laser ablation closure again, and the sinus was closed in 3 months.

**Table 1:** Baseline patients’ characteristics

Characteristic	Value
Sex (male/female)	41/7
Age, years (mean ± SD)	27.7 ± 6.5
BMI, kg/m^2^ (mean ± SD)	23.2 ± 2.7
Preoperative duration of SPD (s), months, median (range)	50.5(32.9,58.3)
Number of pits, median (range)	1.0(0–9)
Previous abscess drainage, *n* (%)	13(27.1)
Duration of length of sinuses (mm) median (range)	34.0(15.0–100.0)
Energy used (J) median (range)	442.9(187.3–1379.3)
Types of SPD, *n* (%)	
Acute SPD in the absence of abscess	17(35.4)
Chronic SPD	31(64.6)
Stage of SPD, *n* (%)	
Stage I	7(14.6)
Stage IIa	8(16.7)
Stage IIb	10(20.8)
Stage III	15(31.2)
Stage IV	7(14.6)
Stage R	1(2.1)

**Table 2:** Postoperative outcome

Variable	Value
Operation time, min (mean ± SD)	15.5 ± 3.3
Patients taking PK ≤ 7 days (%)	3(6.3)
Complications, *n* (%)	
Bleeding	0(0)
Infection/discharge	0(0)
Time, days (mean ± SD)	
To complete healing	27.0 ± 5.5
To walk pain-free	5.0 ± 1.2
To return to work	7.0 ± 3.2
Follow-up, months, median (P_25_,P_75_)	12(3,24)
VAS score, median (P_25_,P_75_)	
Operation day	0(0,2)
Postoperative (1 day)	0(0,2)
Postoperative (3 days)	0(0,1)
Postoperative (7 days)	0(0,1)
Postoperative (14 days)	0(0,0)
Patient satisfaction index *n* (%)	
Very satisfied	46(95.8)
Satisfied	1(2.1)
Neutral	1(2.1)
Dissatisfied	0
Very dissatisfied	0

Abbrev: VAS: visual analog scale

**Table 3:** Healing and recurrence

	Primary healing, *n* (%)	Recurrence, *n* (%)
Types of SPD		
Acute SPD in the absence of abscess (17)	17(100)	1(5.9)
Chronic SPD (31)	31(100)	0(0)
Stage of SPD		
Stage I (7)	7(100)	0(0)
Stage IIa (8)	8(100)	0(0)
Stage IIb (10)	10(100)	0(0)
Stage III (15)	15(100)	0(0)
Stage IV (7)	7(100)	1(14.3)
Stage R (1)	1(100)	0(0)

## Discussion

Mayo first described the disease of SPD in 1830. Although there is no consensus on its optimal treatment, surgery is the only radical cure. Direct resection can be applied, with a cure rate of 85%, a recurrence rate of 20 to 25%, and a more extended recovery period [12, 17, 18]. Skin flap techniques (Karydakis flap technique, Limberg flap transfer technique, “V–Y” flap technique, “Z-shaped” flap technique, etc.) are usually used in more complex or recurrent cases. The operation is complicated due to covering and repairing tissue defects caused by the resection of healthy tissue. The incidence of wound dehiscence ranges from 3 to 15%, increasing the risk of postoperative hematoma and subcutaneous effusion [18–21]. In China, the incidence of this disease is not high, nor is it a common clinical disease; clinicians have some limitations in their understanding of it. However, we usually excise all skin and subcutaneous tissue involved or lap reconstructions; there are differences in the unity of technical operation. We believe that the operation should be minimally invasive, the operation technique should be easy and repeatable, the operation time should be short, painless, the hospital stay should be short, postoperative complications should be prevented, and postoperative recovery should be fast.

In 2014, a study by Handmer [8] demonstrated a minimally invasive sphincter preservation method for treating anal fistula. Suppose an annular laser with a wavelength of 1470 nm is used to treat anal fistula. In that case, the annular energy emitted by the laser catheter acts uniformly on the epithelial cells in the fistula, destroying the epithelial cells and causing the fistula to contract naturally. Dessily et al. [22] applied this technique to SPD. Good results were achieved, and it has been widely used in clinical settings, but no related research has been reported in China. In the latest meta-analysis of laser treatment of pilous sinus by Romic et al. [23], a total of 971 patients were included, with a primary healing rate of 94.4%, a weighted average recurrence rate of 3.8%, and a weighted average complication rate of 10% (*P* < 0.001), all of which were mild complications.

Since the introduction of laser technology in 2017, it has been mainly aimed at hemorrhoids and anal fistula with satisfactory results [24]. Combined with the preliminary experience and existing experience, laser technology is effective in the treatment of Tibetan hair sinus, and the patients feel good after operation. As far as we know, we are the first institution to carry out laser treatment of pilose sinuses in China. In the present study, we found that laser ablation using 1470‐nm radial diode laser fiber was influential in the treatment of SPD. Among the 48 patients, all cases were successfully treated, the cure rate was 100%, and the recurrence rate was 2.1%. This may be related to the preoperative MRI or ultrasonic of all patients to identify the number and branches of sinuses and we exclude the patients in acute abscess stage. During the procedure, we should ensure sufficient drainage. After ablation, secretions or necrotic tissue can be discharged normally to prevent the accumulation of new infection lesions in the tube, improve the success rate of operation, and reduce the risk of recurrence. One patient with recurrent seborrheic dermatitis had a history of seborrheic dermatitis, recurrent skin folliculitis on the shoulder, back, and buttocks, patchy old pigmentation, a long course of disease (2 years), recurrent swelling, and pus in the sacrococcyx. The patient relapsed 3 months after the first laser ablation procedure using 1470‐nm radial diode laser fiber and was treated with the same process after the ineffective drug. The patient was followed up to observe wound healing and amalgamation. In addition, we evaluated the pain degree of the patients during dressing and found that the overall pain of the patients was mild. Patients did not take painkillers. The wounds were minor, and drainage was complete, reducing the stimulation of the postoperative wound inflammatory response and pain. In the present study, the 8 patients in the acute stage in the absence of abscess were not excluded. All patients were cured, indicating that this method is suitable for SPD patients in all stages except acute abscess. However, this conclusion needs to be confirmed in large cohorts.

Compared with other treatments of SPD, the limitation of laser ablation and closure is that it cannot be operated under direct vision, so there is the possibility of omission of sinus branches, incomplete cauterization of epithelial tissue and granulation tissue in the sinus, and incomplete hair cleaning, and these untreated sinus branches and hairs may cause disease recurrence. However, based on our experience, MRI examination before operation and adequate treatment of sinuses during operation can reduce its recurrence. Papagiannopoulos and Zarogoulidis [25] proposed a new method: laser ablation of hair sinuses under the guidance of B-ultrasound during the operation. In their patient cohort, no apparent complications occurred. But in terms of cost, laser technology requires more expensive materials. Nevertheless, the advantages of laser ablation closure are significant, including simple operation, less trauma, mild postoperative pain, and short hospital stay. It has lower requirements for postoperative wound care, fewer postoperative complications, and a lower recurrence rate than traditional surgery. The most important advantage of this technique is that it can enable patients to return to routine work and life quickly, have little impact on employment and study, and improve postoperative satisfaction. Even if relapse occurs after operation, the success rate of reoperation using this technique is 75–78.3% [10, 12].

## Conclusion

Our study shows that laser ablation using 1470‐nm radial diode laser fiber is effective in the treatment of SPD with a low recurrence rate. The surgical technique is minimally invasive and simple straightforward; repeatability. In addition, patients report only mild pain, and nursing requirements are low. This technique has been proposed as a first-line treatment for most patients with SPD, not only for patients in the chronic stage, but also for patients in the acute stage without abscess. This needs to be confirmed by multicenter randomized trials with larger cohorts.

## Figures and Tables

**Fig. 2:**
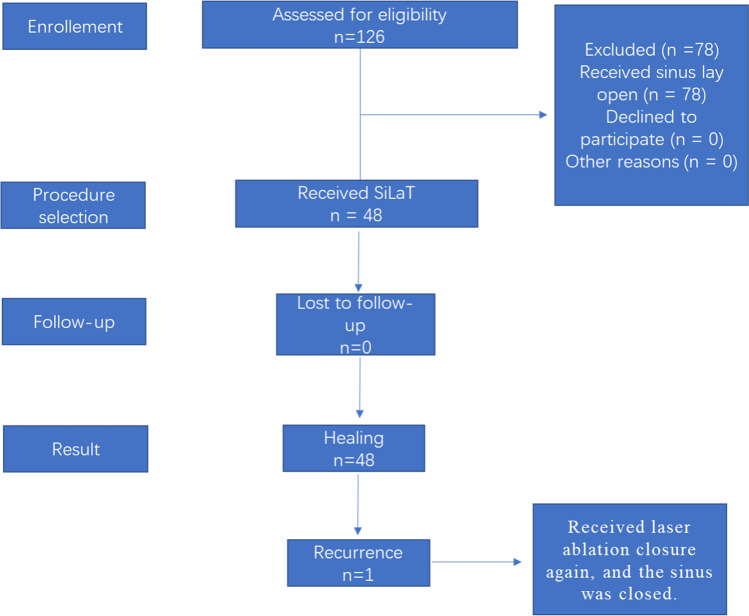
Flowchart demonstrating patients’ selection and exclusion

## Data Availability

Data available on request from the authors.

## Appendix 1

Table 4

**Table 4:** SPD clinical staging system [26]

Stage	Description
Stage I	Single pit in the midline, no lateral extension
Stage II	> 1 pit in the midline, no lateral extension
Stage IIa	2–3 pits in the midline
Stage IIb	> 3 pits in the midline
Stage III	Midline pit/pits plus lateral extension in one direction
Stage IV	Midline pit/pits plus lateral extension in both directions
Stage R	Recurrent SPD following any type of treatment

## Appendix 2

Figure 2
